# The availability of pharmacies in the United States: 2007–2015

**DOI:** 10.1371/journal.pone.0183172

**Published:** 2017-08-16

**Authors:** Dima Mazen Qato, Shannon Zenk, Jocelyn Wilder, Rachel Harrington, Darrell Gaskin, G. Caleb Alexander

**Affiliations:** 1 Department of Pharmacy Systems, Outcomes and Policy, University of Illinois at Chicago, College of Pharmacy, Chicago, Illinois; 2 Division of Epidemiology and Biostatistics, University of Illinois at Chicago School of Public Health, Chicago, Illinois; 3 Department of Health System Sciences, University of Illinois at Chicago, Chicago, Illinois; 4 Department of Health Policy and Management, Johns Hopkins School of Public Health, Baltimore, Maryland; 5 Department of Epidemiology, Johns Hopkins School of Public Health, Baltimore, Maryland; 6 Center for Drug Safety and Effectiveness, Johns Hopkins School of Public Health, Baltimore, Maryland; TNO, NETHERLANDS

## Abstract

**Importance:**

Despite their increasingly important role in health care delivery, little is known about the availability, and characteristics, of community pharmacies in the United States.

**Objectives:**

(1) To examine trends in the availability of community pharmacies and pharmacy characteristics (24-hour, drive-up, home delivery, e-prescribing, and multilingual staffing) associated with access to prescription medications in the U.S. between 2007 and 2015; and (2) to determine whether and how these patterns varied by pharmacy type (retail chains, independents, mass retailers, food stores, government and clinic-based) and across counties.

**Methods:**

Retrospective analysis using annual data from the National Council for Prescription Drug Programs. Pharmacy locations were mapped and linked to the several publically-available data to derive information on county-level population demographics, including annual estimates of total population, percent of population that is non-English speaking, percent with an ambulatory disability and percent aged ≥65 years. The key outcomes were availability of pharmacies (total number and per-capita) and pharmacy characteristics overall, by pharmacy type, and across counties.

**Results:**

The number of community pharmacies increased by 6.3% from 63,752 (2007) to 67,753 (2015). Retail chain and independent pharmacies persistently accounted for 40% and 35% of all pharmacies, respectively, while the remainder were comprised of mass retailer (12%), food store, (10%), clinic-based (3%) or government (<1%) pharmacies. With the exception of e-prescribing, there was no substantial change in pharmacy characteristics over time. While the number of pharmacies per 10,000 people (2.11) did not change between 2007 and 2015 at the national-level, it varied substantially across counties ranging from 0 to 13.6 per-capita in 2015. We also found that the majority of pharmacies do not offer accommodations that facilitate access to prescription medications, including home-delivery, with considerable variation by pharmacy type and across counties. For example, the provision of home-delivery services ranged from less than <1% of mass retailers to 67% of independent stores and was not associated with county demographics, including ambulatory disability population and percent of the population aged ≥65 years.

**Conclusions:**

Despite modest growth of pharmacies in the U.S., the availability of pharmacies, and pharmacy characteristics associated with access to prescription medications, vary substantially across local areas. Policy efforts aimed at improving access to prescription medications should ensure the availability of pharmacies and their accommodations align with local population needs.

## Introduction

In 2015, 4 billion prescriptions or 12.7 prescriptions per-capita, were filled at community pharmacies in the United States [1], and more than 90% of Americans live within 2-miles to one of these pharmacies[2]. Pharmacies are also expanding their operations beyond dispensing to include preventative care, such as health screenings and medication therapy management[3, 4], especially at retail chains. Thus, pharmacies supply and facilitate access to prescription medications and are a vital, and increasingly important, component of healthcare delivery in the United States.

Despite the growing role of pharmacies in healthcare delivery, there is limited information on their prevalence, distribution and characteristics. For example, it is unclear whether there are large differences in the availability of pharmacies across local areas in the U.S., as well as how characteristics of pharmacy operations or accommodations that improve access to prescription medications, such e-prescribing, home delivery, multilingual staffing, and hours-of-operation, vary between different types of pharmacies and local areas. This information is important because accessing and obtaining a prescription medication at a pharmacy is a necessary precondition to adherence, which is known to vary across localities in the U.S. [5, 6].

Several studies suggest that many patients encounter barriers in accessing prescription medications associated with the availability, and characteristics of, their local pharmacies. For example, inadequate pharmacy accessibility[7, 8] or the lack of accommodations, such as home delivery [9] and e-prescribing [10], are associated with higher rates of non-adherence to prescription medications in some communities. There is also some evidence that language concordance between patients and providers, and the provision of multilingual staff or interpreter services at local pharmacies, reduce non-adherence, including primary non-adherence, in non-English speaking patients [11, 12]. Finally, patients filling their prescription medications at independent pharmacies, which are most prevalent in medically underserved areas [7], are less adherent than those using retail chains[8].

We examined trends in the availability of pharmacies and their characteristics in the United States between 2007 and 2015. In addition, we quantified the per-capita availability of pharmacies across counties over time. We were particularly interested in the growth of pharmacies and the provision of accommodations that may promote access to prescription medications, such as multilingual staffing and home delivery. We hypothesized that pharmacies are increasingly available, but that their availability varies substantially across local areas and their characteristics may not always align with the needs of the served population.

## Methods

### Data sources

We used several data sources for this study. First, we derived annual data on community pharmacies in the U.S. from master files provided by the National Council for Prescription Drug Programs (NCPDP) for February 2007 through February 2015 [13]. An NCPDP number is a unique identifier assigned to every licensed pharmacy for processing prescription claims. These data provide detailed information on an annual basis regarding each active community pharmacy in the United States, including location and pharmacy type, which was internally validated by NCPDP. Information on pharmacy characteristics such as home-delivery, is self-reported online by the pharmacy. We geocoded pharmacy addresses using ArcGIS version 10.0.

Second, we used annual estimates from the US Census Bureau’s Population Estimates Program to derive information on total population for all counties for each year [14]. Third, we used estimates from the 5-year (2010–2014) American Community Survey (ACS), the largest national household survey in the United States, to derive information—also at the county level—on the percent of the total adult population 18 years and older) with an ambulatory disability and percent that is non-English speaking defined as those who “speak a language other than English” [15]. We used the 2010 U.S. decennial Census to derive variables on percent of total population 65 and older at the county level [16] Finally, we used data from the Health Resources and Service Administration (HRSA) to identify counties that are designated by the federal government as completely or partially Medically Underserved Areas/Populations (MUA/Ps) or primary health care Health Professional Shortage Areas (HPSA) [17].

We linked annual geocoded information on community pharmacies to county-level data from the American Community Survey, 2010 Census, and HRSA. We included 3,141 counties that comprise the continental U.S. in our analyses.

### Outcome variables

We examined two primary outcomes on an annual basis. First, we quantified pharmacy availability, which we defined as the number of pharmacies overall. We also derived a variable on the number of pharmacies per 10,000 population, or per-capita, using annual county-level estimates on the total population from the Census. To account for changes in the population at the national and county-level over time, we divided the number of pharmacies by the total population for each county each year.

Second, we examined a series of five binary variables for pharmacy characteristics. Pharmacy characteristics were defined as characteristics of pharmacy operations that can improve access to prescription medications. Data for these variables were not available for 2007 and 2008, thus we derived this outcome annually for each pharmacy between 2009 and 2015. In the NCPDP Pharmacy Services Files, pharmacies self-reported whether they “accept electronic prescriptions”, “offer a home delivery service”, “have one or more drive-up windows for prescriptions”, and “offer 24-hour emergency access to a pharmacist with access to the location” [referred to as “24-hour emergency access”]. Pharmacies also reported all the languages spoken by pharmacy staff. Pharmacies reporting a language other than English were coded as offering multilingual pharmacy staff.

### Other variables

We used NCPDP dispenser class codes, such as whether the store was an independent pharmacy or not, and information on any parent organization, such as CVS Health, to classify pharmacies into six mutually exclusive categories: 1) chains, including large retail pharmacies such as Walgreens or Rite Aid; 2) independent (up to three stores under the same parent organization), including franchised pharmacies such as Medicine Shoppe; 3) mass retailers, such as Costco or Target; 4) food stores, such as Giant or Jewel; 5) government, defined as a pharmacy under the jurisdiction of federal, state, county or city government including the Indian Health Service and the Veterans Administration; and 6) clinic-based (retail or government pharmacy located on-site at a clinic, emergency room or outpatient medical center) such as Kaiser Permanente.

### Analysis

First, we used descriptive statistics to examine the distribution of our primary outcomes overall and by pharmacy type aggregated at the national and at county-level. Second, we used time-series ordinary least squares (OLS) linear regression to determine the statistical significance of differences over time in the number of pharmacies per-capita. Third, we used time-series logistic regression to determine the statistical significance in differences in the distribution of pharmacy type and pharmacy characteristics. Fourth, logistic regression was used to compare the prevalence of pharmacy characteristics by pharmacy type for 2015. Fifth, in our county-level analyses, we created categories of quintiles for each primary outcome. To account for differences in the sizes of the total population in different counties, we applied population-weights when deriving national estimates (e.g. mean per-capita, mean % chain, mean % home delivery). We used OLS linear regression to test the statistical significance of the differences in these primary outcomes by quintile category. Sixth, we examined the distribution of select pharmacy characteristics by quintiles of county-level population demographics (i.e. % of total adult population 65 years or older) for 2015. We used OLS linear regression to test the statistical significance of the differences in the mean number of pharmacies per-capita and prevalence of pharmacy characteristics (mean % multilingual staff) by quintile category. Finally, we used the coefficient of variation (COV), calculated as the population-weighted standard deviation divided by population-weighted mean, as a summary measure of the amount of variation in the availability of pharmacies (i.e. mean per-capita), prevalence of pharmacy type (i.e. mean % chain) and pharmacy characteristics (e.g. mean % multilingual) across counties. All analyses were performed using STATA version 14 [College Station, Texas, USA]. The study was considered exempt by the University of Illinois at Chicago Institutional Review Board.

## Results

**Fig 1:**
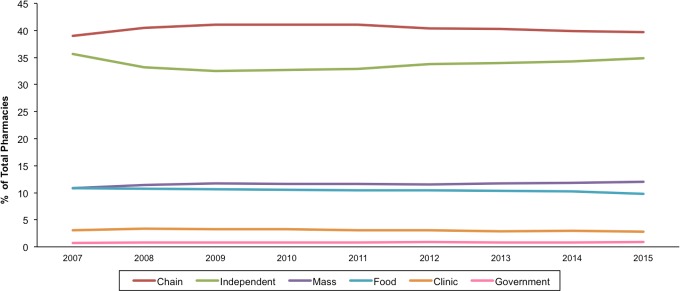
Trends in the availability of community pharmacies by pharmacy type in the US, 2007–2015. Data source: Authors’ analyses of data from the National Council for Prescription Drug Programs on licensed community pharmacies in the U.S. (February 2007-February 2015).

The number of community pharmacies increased by 6.3%, from 63,752 pharmacies in 2007 to 67,753 pharmacies in 2015 (**Table 1**). This trend, however, varied across pharmacy type. For example, there was an 18% increase in mass retailer pharmacies, compared to an 8.3% and 3.8% increase, in chain and independent pharmacies, respectively. The distribution of pharmacy types did not change considerably over this period, with chain and independent pharmacies consistently more prevalent than their counterparts (**Fig 1**). For example, chain pharmacies accounted for 38.8–39.0% of all pharmacies during this period, with fewer accounted for by government-affiliated (~1%), clinic-based (~3%), mass retailer (~11%), food store (~10%) and independent (~35%) pharmacies.

**Table 1:** Total number of pharmacies overall and by pharmacy type, 2007 to 2015.

	2007	2008	2009	2010	2011	2012	2013	2014	2015	% Change (2015 vs. 2007)
**Chain**	24,837	25,424	25,772	26,165	26,429	26,467	26,571	26,632	26,906	8.3%
**Independent**	22,737	20,844	20,389	20,792	21,165	22,124	22,438	22,900	23,596	3.8%
**Mass**	6,901	7,180	7,380	7,429	7,483	7,538	7,717	7,902	8,142	18.0%
**Food**	6,898	6,744	6,710	6,701	6,735	6,838	6,837	6,857	6,636	-3.8%
**Clinic**	1,924	2,078	2,075	2,067	2,003	1,984	1,915	1,957	1,897	-1.4%
**Government**	455	476	493	519	541	562	555	559	576	26.6%
**Overall**	63,752	62,746	62,819	63,673	64,356	65,558	66,033	66,807	67,753	6.3%

Source: Authors analysis of data from the National Council for Prescription Drug Programs (February 2007 to February 2015).

Overall, pharmacy characteristics did not change substantially between 2009 and 2015 (S1 Fig). In 2015, 27% of pharmacies reported offering home-delivery of prescription medications, 18% reported having a drive-up, 12% reported having multilingual staff, and 5% reported having 24-hour emergency access (**Table 2**). By contrast, the share of pharmacies that reported they accept e-prescriptions increased by 40% from 35.8% of all community pharmacies in 2009 to 50.1% in 2015 (p<0.001).

**Table 2:** Pharmacy characteristics overall and by pharmacy type, 2015.

	Chain	Independent	Mass	Food	Clinic	Government	Overall
**24 hour emergency access, %**	10.7	1.2*	0.85*	0.73*	2.24*	7.5*	**4.9**
**Drive-up, %**	23.9	15.5*	2.64*	21.0	2.2*	4.9*	**17.5**
**Home Delivery, %**	6.24	66.6*	0.02	9.25*	24.9*	7.8	**27.1**
**Multilingual Staff, %**	1.8	30.6*	0.2	1.54	28.7*	6.9*	**12.4**
**Accept e-prescriptions, %**	49.2	56.7*	28.4*	61.2*	43.1*	13.6*	**50.1**

Source: Authors analysis of data from the National Council for Prescription Drug Programs (February 2015). Notes: Logistic regression was used to compare the prevalence pharmacy accommodations across pharmacy types. Significance refers to differences in prevalence between chain and all other pharmacy types.

*p-<0.001

There were large differences in pharmacy characteristics by pharmacy type (**Table 2**). Of the 26,789 chain pharmacies in 2015, 10.7% were opened for 24 hours and 23.9% reported offering a drive-up window, which is a significantly higher proportion than all other pharmacy types. Chain pharmacies, however, were significantly less likely than independent pharmacies to report offering home-delivery (6.2% vs. 64.2%) or multilingual staff (1.8% vs. 30.6%). All of these differences were statistically significant (p<0.001). This distribution of these characteristics by pharmacy type was similar in 2009.

**Fig 2:**
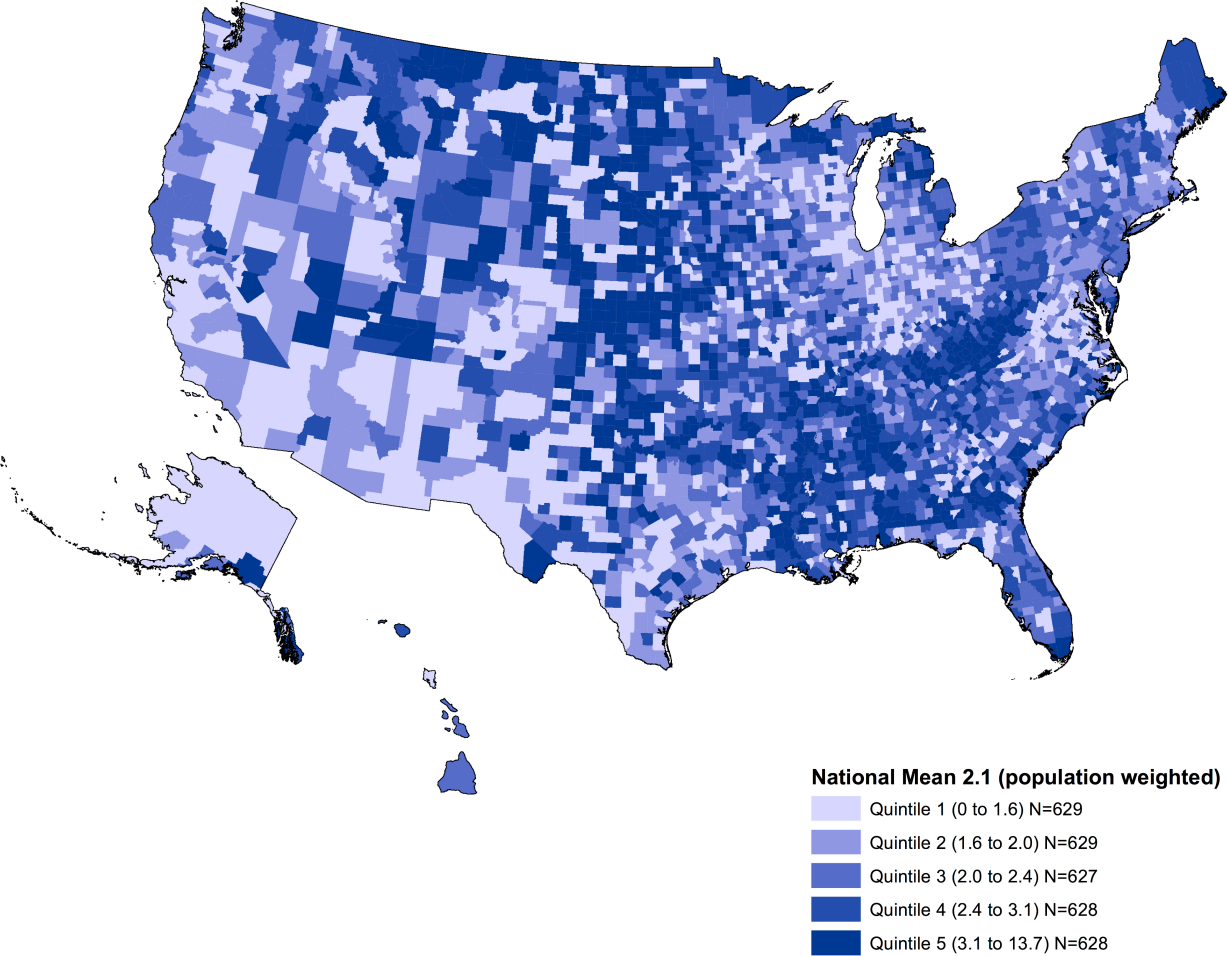
Pharmacies per 10,000 People by County in the U.S., 2015. Data source: Authors’ analyses of data from the National Council for Prescription Drug Programs on licensed community pharmacies in the U.S. for February 2015, and the US Census Bureau’s Population Estimates Program to derive information on annual total population for all counties (N = 3,141) for each year between 2007 and 2015.

Although the number of pharmacies per-capita remained at 2.11 per 10,000 individuals between 2007 and 2015 (S2 Fig), we found substantial variation across counties (**Fig 2**). In 2015 counties in the highest quintile had nearly three-fold more pharmacies than those in the lowest quintile (mean 3.6 vs. 1.3 per-capita, respectively; p<0.001 from regression). Counties in the lowest quintile appear to cluster in the Pacific West, Southwest and Great Lakes regions, while counties with the highest tend to be located in the Northeast, Southeast, Northern Appalachia, and Plain states.

**Fig 3:**
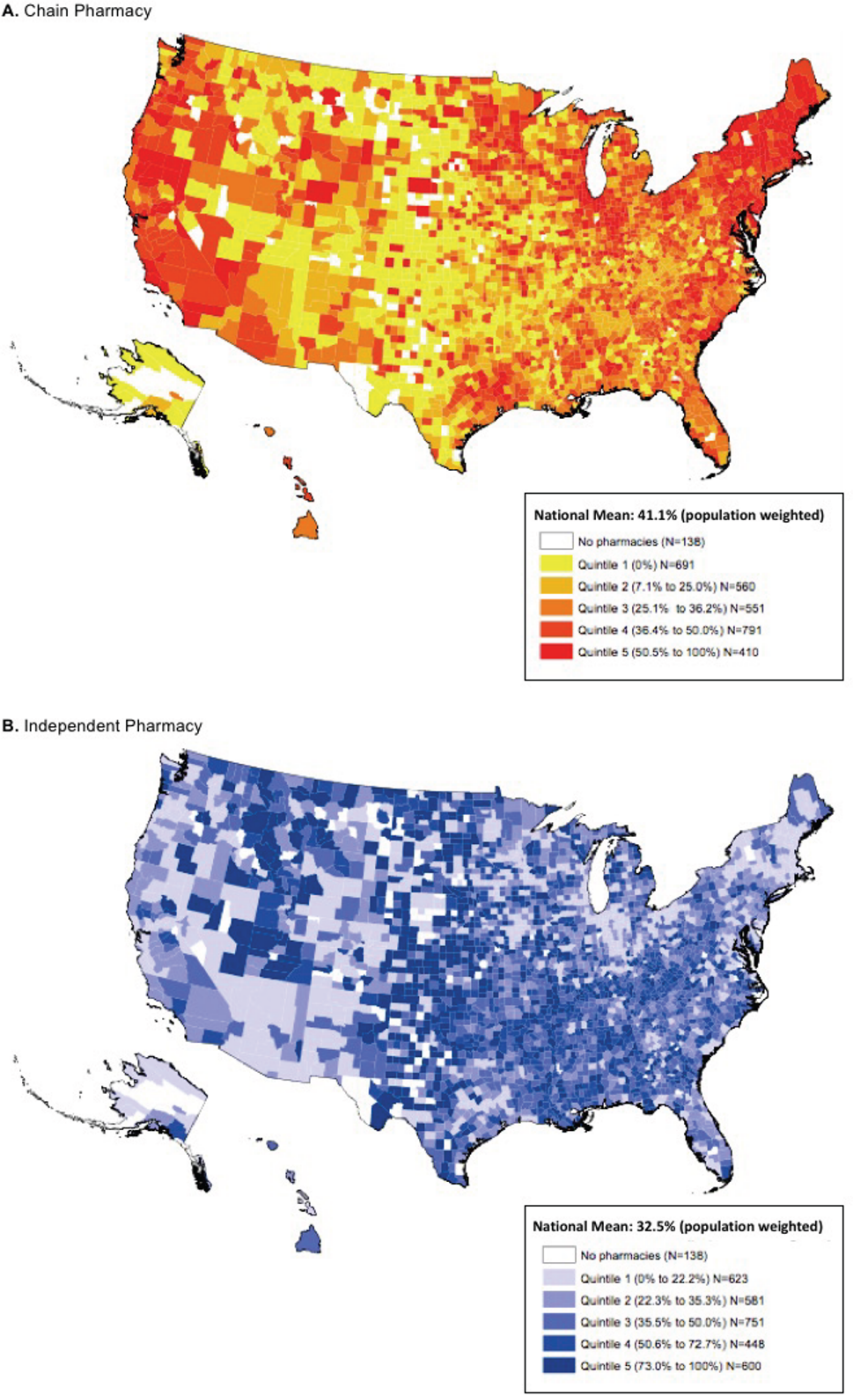
Retail Chain and Independent Pharmacies by County in the U.S., 2015.

**Fig 3**depicts the distribution of chain and independent pharmacies across counties in 2015. Chain pharmacies predominate in the Northeast and Western regions, while independent pharmacies account for the majority of pharmacies in the Southeast, Southwest and Plain states. Independent pharmacies accounted for the total pharmacy market in 446 counties (14% of counties); more than half (52.3%) of these counties, however, had only one independent pharmacy. In contrast, less than 2% (66 counties) were exclusively comprised of retail chains. In addition, there are disproportionately more independent pharmacies in counties designated as MUAs (35.6%) when compared to non-MUAs (30.6%) (p<0.001).

**Fig 4:**
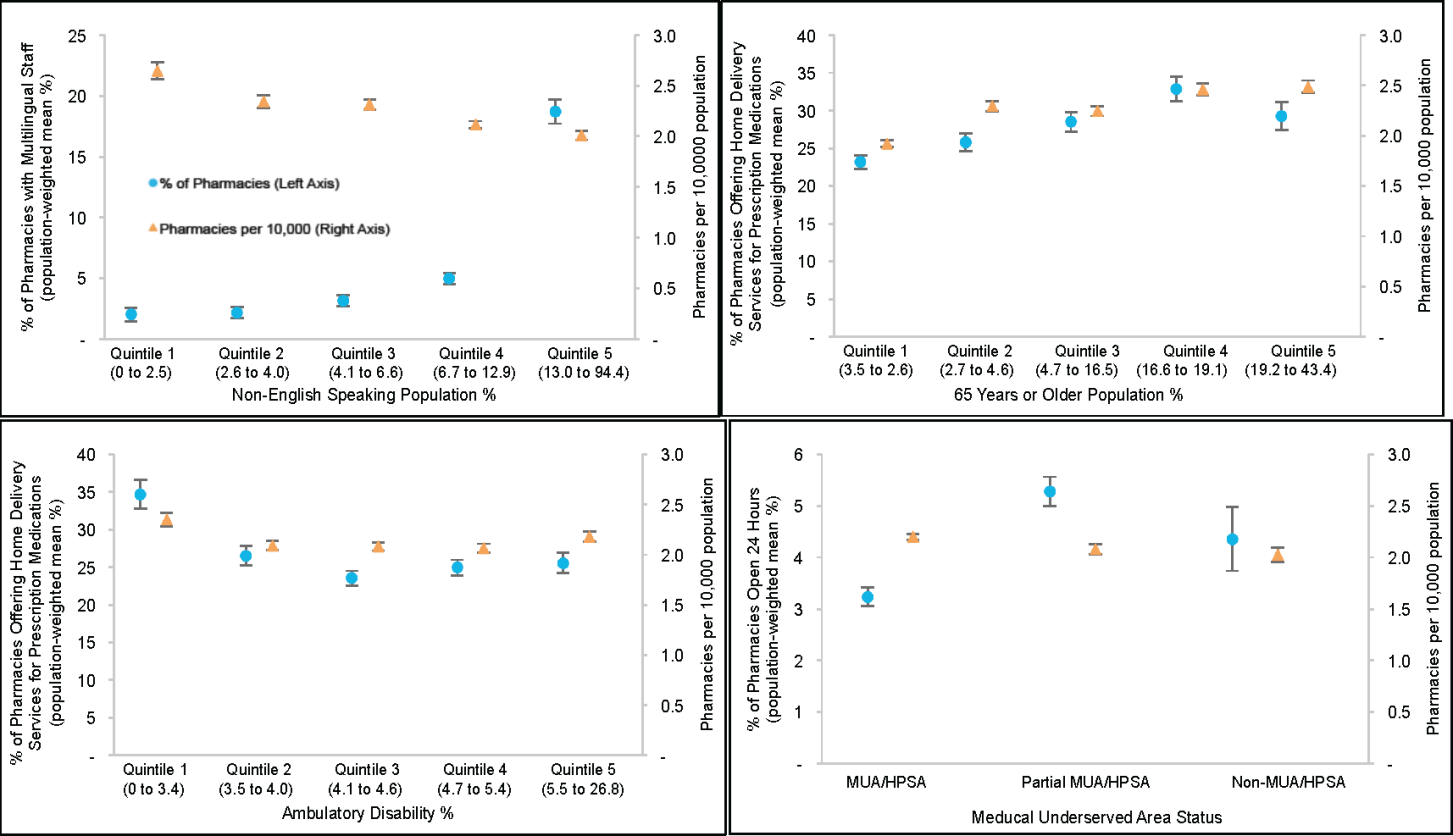
The availability of pharmacies and accommodations by quintiles of county population demographics, 2015. Data sources: Authors’ analyses of data from the National Council for Prescription Drug Programs on licensed community pharmacies in the U.S. for February 2015; the US Census Bureau’s Population Estimates Program to derive information on annual total population at the county-level for 2015; 2010 US decennial Census to derive information in the % of the population aged 65 years or older; 5-year estimates (2010–2014) from the American Community Survey to derive information on the percent of the population that is non-English speaking defined as those who “speak a language other than English” and percent of the adult population that has an ambulatory disability; and the Health Resources and Services Administration to identify counties that are designated as completely or partially Medically Underserved Areas/Populations (MUA/P) or primary care Health Professional Shortage Areas (HPSA). Quintiles (range) are reported from lowest to highest percent (Quintiles 1 to 5) of county population demographics. For example, 0 to 2.5% of the total population is non-English speaking for counties in Quintile 1. Reported means are population-weighted to account for differences in the size of county total populations; Error-Bars are 95% Confidence Intervals.

**Fig 4**depicts the association between the per-capita availability of pharmacies, select pharmacy characteristics and population demographics at the county level. Although there was no substantial difference in the availability (or accessibility) of pharmacies by MUA status, pharmacies offering 24-hour emergency access were least prevalent in MUAs. There were also notable differences across population demographics. For example, counties in the highest non-English speaking population quintile had significantly fewer pharmacies than those in the lowest quintile (1.97 vs. 2.57 per-capita, respectively; p<0.001). Although multilingual pharmacies were most prevalent in these counties, more than 80% of pharmacies in these areas did not have multilingual staff in 2015. The share of pharmacies offering home-delivery of prescription medications was less in counties with the highest population of adults with an ambulatory disability compared to those counties with the least (25.5% vs. 34.7%, respectively; p<0.001). In addition, there was no strong association between the proportion of pharmacies offering home delivery and quintiles for percent of the population 65 years or older.

Pharmacy characteristics also varied substantially across counties. This variation was particularly pronounced for multilingual staffing and least pronounced for e-prescribing (coefficient of variation 1.02 vs. 0.26, respectively). (S3 Fig).

## Discussion

We linked detailed information from the National Council for Prescription Drug Programs with publically available demographic data to examine the availability and characteristics of pharmacies in the United States. The total number of pharmacies increased by 6.3% over a nine-year period, reaching more than sixty-seven thousand pharmacies in 2015. The availability of pharmacies per-capita, however, did not change during this period, but varied substantially across local areas. While retail chains persistently account for the largest share of the pharmacy market, independent pharmacies continued to constitute approximately a third of all stores in the U.S. With the exception of e-prescribing, there was no marked change in pharmacy characteristics, including accommodations that may promote access to prescription medications for vulnerable Americans, such as home delivery and multilingual staffing.

To our knowledge, this is the first study to characterize the availability of pharmacies at the national and local level. This information is important to a variety of stakeholders in both the public and private sector, including local, state and federal public health and policy officials and pharmacy retailers, interested in better understanding the role of pharmacies in improving access and adherence to prescription medications. Although policy efforts—such as Medicare Part D—have focused on ensuring the affordability of prescription medications [18], non-adherence, which varies across localities [5, 6], persists as important public health problem in the U.S. [19], suggesting access barriers, including pharmacy accessibility and the provision of pharmacy accommodations associated with access to prescription medications, are also important to consider.

Despite the growing number of pharmacies in the U.S., we identified nearly a three-fold difference in their availability across local areas. This extends findings on geographic variation in access to care [20–22], to pharmacies. In 2015, there was more than a 3-fold difference in the number of pharmacies per-capita between counties in the highest and those in the lowest quintile, with no clear difference by MUA status. Specifically, there were fewer pharmacies located in the Southwest and Pacific West regions of the country, including counties in Texas, California, New Mexico and Arizona; many of these areas also have a disproportionately higher rate of medication non-adherence among Medicare-Part D beneficiaries[5]. These findings suggest some localities are disproportionately more likely to encounter barriers in the availability of pharmacies when attempting to fill and adhere to their prescription medications.

Although the distribution of pharmacies by type has not changed over time, it varies across local areas. While retail chains dominate the pharmacy market, and fewer than one-fifth of prescriptions are dispensed at independent pharmacies [23], independent pharmacies persistently accounted for more than one-third of all community pharmacies in the U.S. In fact, in numerous areas in the country, particularly in the Southwest and Plain states, independents dominate market share and are frequently the only pharmacy serving the local population. Ensuring pharmacies are available and accessible in these populations should be a public health priority considering independents are the most at-risk for pharmacy closures [24].

According to our analyses of pharmacy characteristics, the provision of accommodations that may improve access to prescription medications has not changed and the vast majority of pharmacies do not offer them. For example, only one-fourth of pharmacies offered home delivery, despite a growing population of homebound elderly [25], as well as some evidence that home delivery improves medication adherence [9]. The availability of 24-hour pharmacies is also of interest; since only one in twenty pharmacies we examined are opened for 24-hours, yet longer hours of operation may be associated with lower hospital re-admissions [26]. Despite federal legislation that mandates non-discriminatory access to accommodations and language services [27], our findings also suggest that many pharmacies lack multilingual staffing which may impede access and adherence to prescription medications for a growing population of immigrant Americans who may not be proficient in English [28].

While we found a large increase in the share of pharmacies that reported accepting e-prescriptions, nearly half of pharmacies still didn’t accept them in 2015. This is surprising considering most pharmacies are enabled for, and seventy percent of clinics have adopted, e-prescribing [29]. The underuse of e-prescribing by pharmacies may be due to the exclusion of pharmacies as eligible providers under the Centers for Medicare and Medicaid Services electronic health record incentive programs, for which e-prescribing is part of the “meaningful use” requirement [30]. Including pharmacies as eligible providers may encourage pharmacy participation and promote the “meaningful use” of e-prescribing and, most importantly, reduce medication non-adherence [9].

We also found that pharmacy characteristics, specifically the provision of accommodations associated with access to prescription medications, varied across counties and may not align with the needs of the local population. For example, multilingual pharmacies were only slightly more prevalent in predominately non-English speaking counties. For example, more than 90% of the population in Starr County, Texas speaks a language other than English, but only six of the twelve pharmacies have multilingual staff. In addition, less than one-third of pharmacies located in counties with a disproportionately higher older adult or ambulatory disability population offered home-delivery services. These findings suggest Americans that do not speak English and the homebound elderly may encounter accommodation, including language, barriers as they attempt to fill their prescription medications or engage with community pharmacies.

Efforts to improve access to pharmacies, and, in turn, prescription medications, should consider policies and programs that support the measuring and monitoring of pharmacy accessibility. For example, the Health Resources and Services Administration (HRSA), the federal agency responsible for designated MUA/Ps and health professional shortage areas, should consider including a designation to identify pharmacy shortage areas. In partnership with pharmacy retailers, federal and state policy officials and local health departments can then prioritize resources and funding decisions to target the development of pharmacies in these pharmacy shortage areas.

Pharmacy retailers, including chains and independents, may also consider monitoring population demographics to inform decisions on pharmacy operations to better ensure pharmacies offer accommodations that specifically target the needs of the local population. For example, the provision of home-delivery services would be a priority for pharmacies located in areas that have a disproportionately higher homebound elderly population. Such efforts can strengthen the capacity of pharmacies to promote access to prescription medications locally, particularly in vulnerable populations, and also support a more efficient and equitable distribution of pharmacy accommodations.

Our analyses have several limitations. First, our information regarding the characteristics of pharmacies is based on self-report. However, we randomly selected a subset of 100 pharmacies and were able to independently validate that 98 were operational. Of these 100 cases, the data we obtained regarding 24-hour emergency access and drive-up service were correct in 95 and 98% of cases, respectively. Our national findings on the prevalence of home-delivery services among independent pharmacies and the percent of pharmacies that accept e-prescriptions were similar to prior reports [29, 31]. In addition, our information is comparable to our prior analyses of pharmacy licensure in one large Midwestern city [7]. Second, while we were able to assess important pharmacy characteristics that may impact access to prescription medications, these characteristics nevertheless provide an incomplete picture of how easily consumers can use these pharmacies. For example, we do not incorporate the hours of operation of these stores, walkability, vehicle ownership, public transportation characteristics of counties, and geographic accessibility based on travel distance/time to nearest pharmacy. We also do not capture information on interpreter services, which may be offered at pharmacies that lack multilingual staff. Third, there may be considerable variation in the availability of pharmacies within counties [7]. Fourth, the target population served by pharmacies we have defined as government or clinic-based, in contrast to retail pharmacies, may not include the entire local population. Finally, while we characterized how pharmacy characteristics varied based on several county characteristics, we did not incorporate information that directly measures the need for pharmacies to be located in a specific geography (e.g. demand for prescription medications based on underlying disease burden).

## Conclusion

Although the number of pharmacies has slightly increased over the last nine years in the United States, with both retail chains and independent pharmacies consistently leading the pharmacy market, availability of pharmacies varies substantially across local areas. Many pharmacies do no offer accommodations that facilitate access to prescription medications, and pharmacists, and, in turn, promote medication adherence. Future programs and policies should address the availability of pharmacies and ensure pharmacy characteristics, including accommodations such as multilingual staffing and home delivery, align with local population needs.

## Supporting information

S1 Fig Trends in pharmacy characteristics and accomodation services overall and by pharmacy type, 2009–2015. (PDF)

S2 Fig Number of pharmacies per 10,000 population (per-capita) in the U.S., 2007–2015. (PDF)

S3 Fig The availability of pharmacy accomodations by county in the U.S., 2015. (PDF)
