# Supplementary figures and images for: The availability of pharmacies in the United States: 2007–2015
Source: PLoS One. 2017 Aug 16;12(8):e0183172. doi: 10.1371/journal.pone.0183172 (PMC5559230; doi:10.1371/journal.pone.0183172)

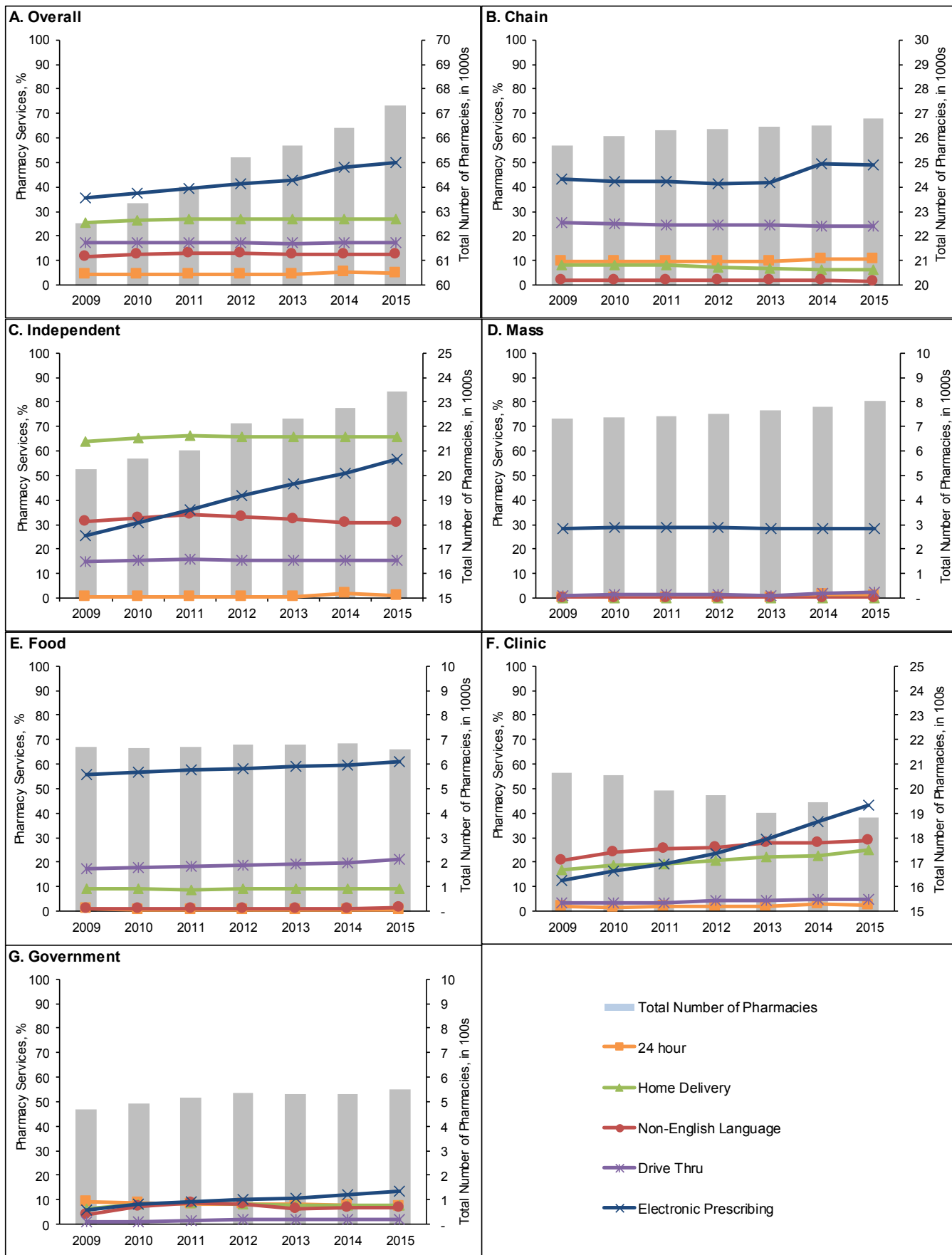

Supplement: S1 Fig — (PDF) [file pone.0183172.s001.pdf]

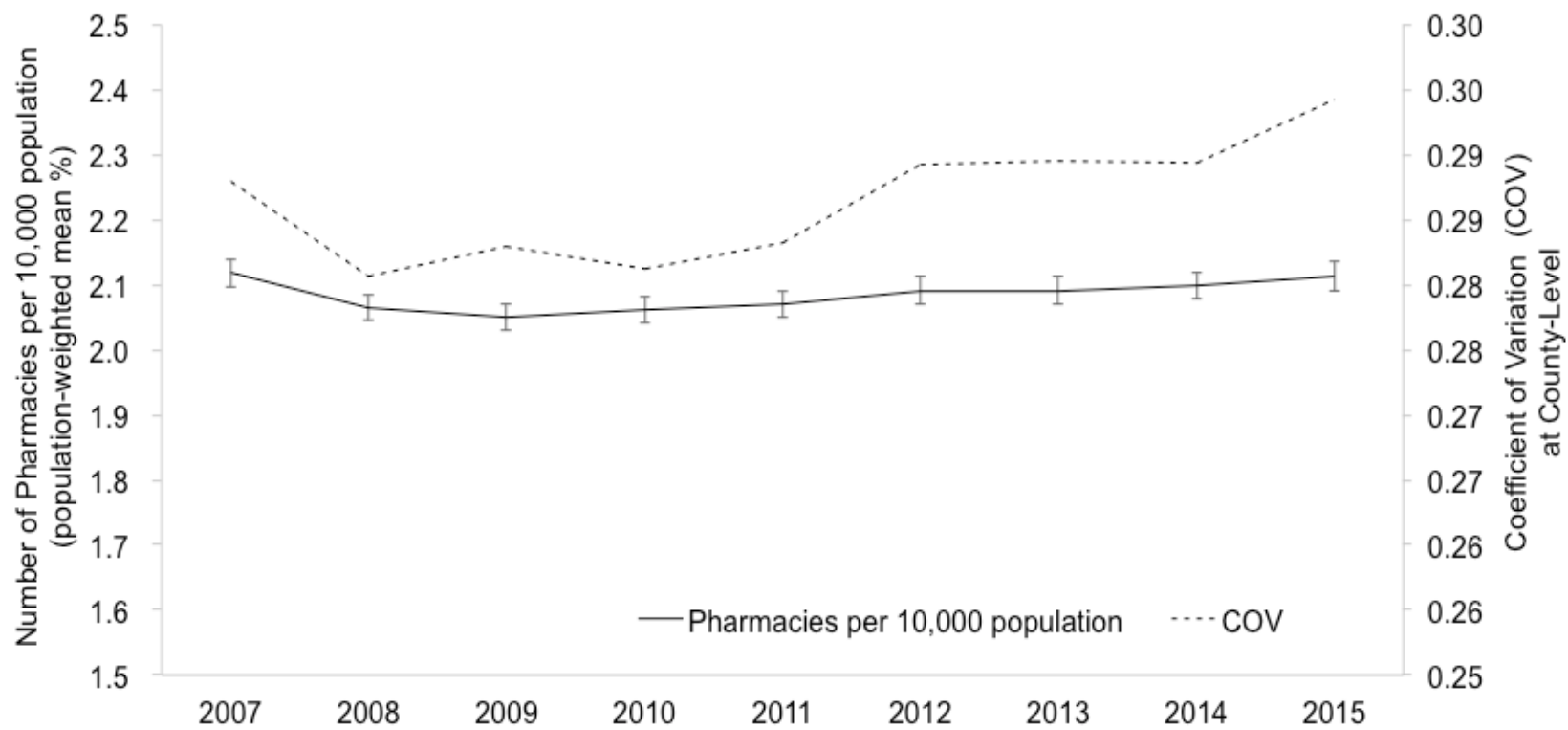

Supplement: S2 Fig — (PDF) [file pone.0183172.s002.pdf]
