# Supplementary material for: The availability of pharmacies in the United States: 2007–2015
Source: PLoS One. 2017 Aug 16;12(8):e0183172. doi: 10.1371/journal.pone.0183172 (PMC5559230; doi:10.1371/journal.pone.0183172)
Supplement: S3 Fig — (PDF) [file pone.0183172.s003.pdf]

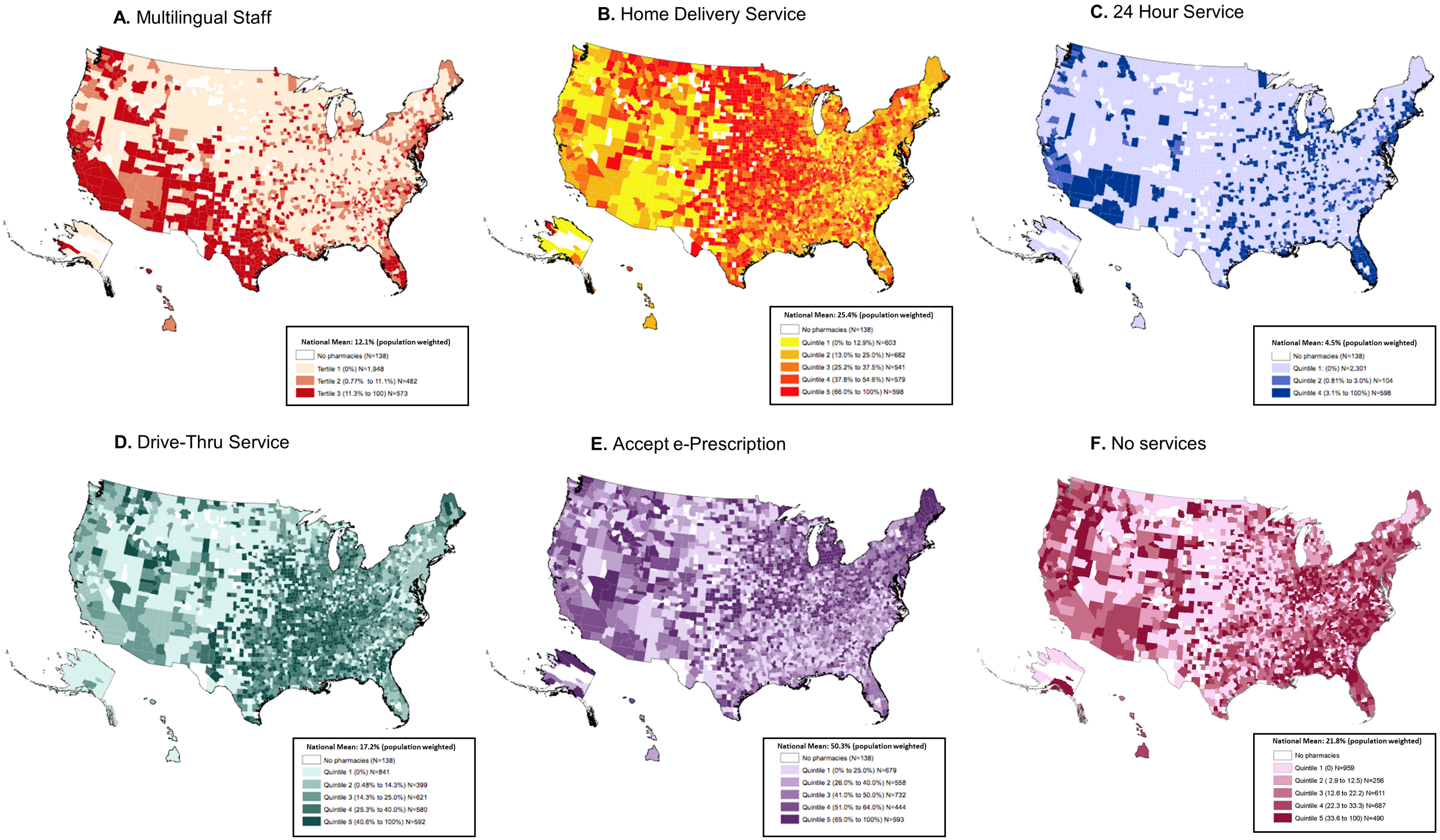

A. Multilingual Staff
National Mean: 12.1% (population weighted)
No pharmacies (N=138)
Tertile 1 (0%) N=1,948
Tertile 2 (0.77% to 11.1%) N=482
Tertile 3 (11.3% to 100) N=573
B. Home Delivery Service
National Mean: 25.4% (population weighted)
No pharmacies (N=138)
Quintile 1 (0% to 12.9%) N=603
Quintile 2 (13.0% to 25.0%) N=682
Quintile 3 (25.2% to 37.5%) N=541
Quintile 4 (37.8% to 54.6%) N=579
Quintile 5 (66.0% to 100%) N=598
C. 24 Hour Service
National Mean: 4.5% (population weighted)
No pharmacies (N=138)
Quintile 1: (0%) N=2,301
Quintile 2 (0.81% to 3.0%) N=104
Quintile 4 (3.1% to 100%) N=598
D. Drive-Thru Service
National Mean: 17.2% (population weighted)
No pharmacies (N=138)
Quintile 1 (0%) N=841
Quintile 2 (0.48% to 14.3%) N=399
Quintile 3 (14.3% to 25.0%) N=621
Quintile 4 (25.3% to 40.0%) N=580
Quintile 5 (40.6% to 100%) N=592
E. Accept e-Prescription
National Mean: 50.3% (population weighted)
No pharmacies (N=138)
Quintile 1 (0% to 25.0%) N=679
Quintile 2 (26.0% to 40.0%) N=558
Quintile 3 (41.0% to 50.0%) N=732
Quintile 4 (51.0% to 64.0%) N=444
Quintile 5 (65.0% to 100%) N=593
F. No services
National Mean: 21.8% (population weighted)
No pharmacies
Quintile 1 (0) N=959
Quintile 2 ( 2.9 to 12.5) N=256
Quintile 3 (12.6 to 22.2) N=611
Quintile 4 (22.3 to 33.3) N=687
Quintile 5 (33.6 to 100) N=490
